# Supplementary material for: Mamestra brassicae Multiple Nucleopolyhedroviruses Prevents Pupation of Helicoverpa armigera by Regulating Juvenile Hormone Titer
Source: Insects. 2024 Mar 18;15(3):202. doi: 10.3390/insects15030202 (PMC10971710; doi:10.3390/insects15030202)
Supplement: Supplementary file 1 [file insects-15-00202-s001.zip › insects-2844554-supplementary.pdf]

Table S1. Primers used for real-time PCR.

Names	Sequences (5′–3′)
MbMNPV poly-F	CGTAAACTGGAGCGGTAAAG
MbMNPV poly-R	GACTTCGTGGGGCACATAG
Hear β-actin-F	CACCGCTGAGAGGGAAATC
Hear β-actin-R	CATACCCAGGAAGGAAGGC
JHE-F	GAATACGACAAAAATCCCCG
JHE-R	CCTGTTCCGCTCATCAAAAT
selenbp1-a-F	CCTCCTTATCCTGCCTGCT
selenbp1-a-R	CGTCTTTATCTCCCATCGTCG
bhmt-F	CTTAGAGCGGGAGCAGATTT
bhmt-R	CACATAGTCCTGGTACTCCTCG
JHEH-F	GTTTCCTTGTAAAAGCGGTG
JHEH-R	GCCTGATGCTCTGATCCTGT
ACADSB-F	ATGTTCCCATTACGCCGC
ACADSB-R	CGCCAGTTTTCTGATTGTGTCT

Table S2. Primers used for synthesizing dsRNA.

Names	Sequences (5′–3′)
JHE-F-CDS	AGGTGCCCGACACCGCACA
JHE-R-CDS	CCGCTGAACCCTTAACTATTAACAACA
JHEH-F-CDS	ATGGGTTTCCTTGTAAAAGCG
JHEH-R-CDS	GTCACAGTTCAGTCTTCTTGTTCT
JHE-F-T7	TAATACGACTCACTATAGGGGAATACGACAAAAATCCCCG
JHE-R-T7	TAATACGACTCACTATAGGGGACTCTTTCAAATCCATCACTAGC
JHEH-F-T7	TAATACGACTCACTATAGGGTGGTTTGCCTTCAAGTGCT
JHEH-R-T7	TAATACGACTCACTATAGGGGCCGGTTCATCAGATTCC
EGFP-F-T7	TAATACGACTCACTATAGGGGTGCAGTGCTTCAGCCGCTA
EGFP-R-T7	TAATACGACTCACTATAGGGTCCATGCCGTGAGTGATCCC
